# Acetaminophen attenuates lipopolysaccharide-induced cognitive impairment through antioxidant activity

**DOI:** 10.1186/s12974-016-0781-6

**Published:** 2017-01-21

**Authors:** Wei-Xing Zhao, Jun-Han Zhang, Jiang-Bei Cao, Wei Wang, Dong-Xin Wang, Xiao-Ying Zhang, Jun Yu, Yong-Yi Zhang, You-Zhi Zhang, Wei-Dong Mi

**Affiliations:** 1 Anesthesia and Operation Center, Chinese PLA General Hospital, 28th Fuxing Road, Haidian District, Beijing, 100853 China; 2 The Second Affiliated Hospital of Xiangya School of Medicine, Central South University, Changsha, 410008 China; 3 Department of Anesthesiology, The General Hospital of the PLA Rocket Force, Beijing, 100088 China; 4 Department of Anesthesiology and Surgical Intensive Care, Peking University First Hospital, Beijing, 100034 China; 5 Institute of Pharmacology and Toxicology, Beijing Key laboratory of Neuropsychopharmacology, 27th Taiping Road, Haidian District, Beijing, 100850 China

**Keywords:** Acetaminophen, Neuroprotective therapy, Antioxidant activity, Oxidative stress, Neuroinflammation, Memory impairment, Apoptosis

## Abstract

**Background:**

Considerable evidence has shown that neuroinflammation and oxidative stress play an important role in the pathophysiology of postoperative cognitive dysfunction (POCD) and other progressive neurodegenerative disorders. Increasing evidence suggests that acetaminophen (APAP) has unappreciated antioxidant and anti-inflammatory properties. However, the impact of APAP on the cognitive sequelae of inflammatory and oxidative stress is unknown. The objective of this study is to explore whether APAP could have neuroprotective effects on lipopolysaccharide (LPS)-induced cognitive impairment in mice.

**Methods:**

A mouse model of LPS-induced cognitive impairment was established to evaluate the neuroprotective effects of APAP against LPS-induced cognitive impairment. Adult C57BL/6 mice were treated with APAP half an hour prior to intracerebroventricular microinjection of LPS and every day thereafter, until the end of the study period. The Morris water maze was used to assess cognitive function from postinjection days 1 to 3. Animal behavioural tests as well as pathological and biochemical assays were performed to evaluate LPS-induced hippocampal damage and the neuroprotective effect of APAP.

**Results:**

Mice treated with LPS exhibited impaired performance in the Morris water maze without changing spontaneous locomotor activity, which was ameliorated by treatment with APAP. APAP suppressed the accumulation of pro-inflammatory cytokines and microglial activation induced by LPS in the hippocampus. In addition, APAP increased SOD activity, reduced MDA levels, modulated glycogen synthase kinase 3β (GSK3β) activity and elevated brain-derived neurotrophic factor (BDNF) expression in the hippocampus. Moreover, APAP significantly decreased the Bax/Bcl-2 ratio and neuron apoptosis in the hippocampus of LPS-treated mice.

**Conclusions:**

Our results suggest that APAP may possess a neuroprotective effect against LPS-induced cognitive impairment and inflammatory and oxidative stress via mechanisms involving its antioxidant and anti-inflammatory properties, as well as its ability to inhibit the mitochondrial permeability transition (MPT) pore and the subsequent apoptotic pathway.

## Background

Postoperative cognitive dysfunction (POCD) is characterized by a decline in cognitive function that occurs in patients after anaesthesia and surgery when compared to their preoperative cognitive status [1]. The decreased cognitive functions include impairments in working memory, long-term memory, information processing, attention and cognitive flexibility [2, 3]. The incidence of POCD indicates increased risk of mortality, decreased quality of life, risk of early withdrawal from the workforce and increased dependency [4]. There is emerging evidence that neuroinflammation is implicated in the pathophysiology of POCD and other progressive neurodegenerative disorders such as Alzheimer’s disease (AD), Parkinson’s disease, amyotrophic lateral sclerosis (ALS) and multiple sclerosis (MS) [5–7]. Evidence has also shown that oxidative stress is harmful to cognitive function and is thought to contribute to the pathogenesis of the above neurodegenerative diseases [8–10]. In fact, neuroinflammation and oxidative stress exist simultaneously, and the interaction between oxygen free radicals and inflammatory factors aggravates cognitive deficiency [11, 12]. However, the exact pathogenesis underlying the effect of neuroinflammation and oxidative stress on cognitive function is still unclear.

Lipopolysaccharide (LPS) is a major bacterial TLR4 ligand that activates the innate immune response to infections [13]. Intracerebroventricular administration of LPS in mice produces cognitive impairment by expression of pro-inflammatory cytokines and neuronal death via apoptosis [14–19], and oxidative stress induced by LPS has been implicated in memory impairment [20]. This method can be effectively used as an animal model for POCD [15, 19].

Acetaminophen (*N*-acetyl-4-aminophenol), also known as APAP or paracetamol, is one of the most widely used medications all over the world. APAP exhibits both analgesic and antipyretic properties and has been widely used as an active ingredient in many approved drugs. Additionally, since the 1980s, APAP has become the first drug of choice for the treatment of pain and fever in children due to the high incidence of Reye’s syndrome associated with aspirin [21]. According to the US Food and Drug Administration (FDA), 479 drugs contain APAP. As of August 2011, 235 out of the 479 APAP-containing drugs have an active approval status, including 214 prescription drug products owned by 31 companies and 21 over-the-counter (OTC) drug products produced by nine companies [22]. Given its wide use and easy availability, scientists have recently begun to examine APAP for off-label applications [22].

It has also been reported that APAP has neuroprotective effects. Maharaj et al. [23] reported that APAP (0.25–1 mM) treatment ex vivo can inhibit cyanide-induced superoxide anion generation and lipid peroxidation in rat brain homogenates. Further animal study has suggested that APAP (100 mg/kg/day, i.p.) can inhibit quinolinic acid (QA)-induced lipid peroxidation, superoxide anion generation, and cell damage in the rat hippocampus [24]. Tripathy et al. [25, 26] showed that low-dose APAP reduces inflammatory protein release from cultured brain neuronal and endothelial cells exposed to oxidant stress and increases expression of the anti-apoptotic protein Bcl-2 in brain neurons. Naziroglu et al. [27] also reported that APAP (5–100 mg/kg) can reduce brain and microsomal lipid peroxidation, while it also increases brain vitamin E levels and microsomal glutathione peroxidase (GSH-Px) activity. In addition, APAP has been shown to protect dopaminergic neurons from oxidative damage evoked by acute exposure to 6-hydroxydopamine or excessive levels of dopamine in vitro [28], suggesting a potential benefit for PD. Finally, recent studies have shown that APAP shows nootropic activity through increasing the escape latency in the step through passive avoidance paradigm task and decreasing acetyl cholinesterase activity in colchicine-induced cognitive impairment (an animal model for AD) in rats [29], suggesting a possible therapeutic effect of APAP in AD.

Based on the above findings, we hypothesized that during neuroinflammation, APAP might attenuate oxidative stress and inflammatory cytokines in the hippocampus and thus improve cognitive impairment. To test this hypothesis, we assessed the neuroprotective effects of APAP against LPS-induced neuroinflammation and oxidative damage in the present study. The results obtained in this study may provide new insight into the mechanism of APAP for the treatment of cognitive impairment.

## Methods

### Animals

Adult male C57BL/6 (*n* = 80) mice aged 10–11 weeks and weighing 21–23 g were purchased from Vital River Laboratories Animal Technology Co. Ltd. (Beijing, China. Permit Number: SCXK(JING) 2012-0001). All mice were housed in groups of 3 to 5 per plastic cage (24 × 36 × 24 cm) and given free access to standard food and water in an air-conditioned room set at 24 ± 1 °C with 50 ± 10% humidity, under a standard 12–12 light-dark cycle (lights on 7 AM to 7 PM). The animals were acclimatized for 7 days before the experiment and were group-housed with the same cage mates throughout the acclimation and testing periods. The procedures on animal experimentation were approved by the Animal Care Committee of the Chinese People’s Liberation Army General Hospital (Beijing, China). The maintenance and handling of the mice were consistent with the guidelines of the National Institutes of Health, and adequate measures were taken to minimize animal discomfort.

### Drug treatment

The mice were divided into four groups randomly (20 mice per group): the control plus placebo group (CON group), control plus APAP group (APAP group), LPS plus placebo group (LPS group) and the LPS plus APAP group (A + L group). The LPS-induced cognitive impairment mouse model was performed according to a protocol previously described by our laboratory [19]. LPS was administered via the intracerebroventricular (i.c.v.) route. Each mouse was anaesthetized with chloral hydrate (400 mg/kg, i.p.). LPS (Sigma, St. Louis, MO, USA), dissolved in artificial cerebrospinal fluid (aCSF: 140 mM NaCl, 3.0 mM KCl, 2.5 mM CaCl_2_, 1.0 mM MgCl_2_ and 1.2 mM Na_2_HPO_4_) with pH = 7.4 (2 μg in 2 μL), was infused into the lateral ventricle using a mouse brain stereotactic apparatus (Kopf Instruments, Tujunga, CA, USA). The stereotactic coordinates from bregma were derived according to Zhang [19]. The coordinates were 0.5 mm caudal to bregma, 1.0 mm right lateral to the sagittal suture and 2.0 mm ventral of the dura. Using a 10-μL microsyringe, the injection speed was set at 0.667 μL/min and the needle was held in place for 2 min for proper dispersal of the drug from the tip following injection.

In mice in the CON group and APAP group, 2 μL of aCSF was infused into the lateral ventricle. Mice in the LPS group and A + L group were infused with LPS. Animals in the APAP group and A + L group received intraperitoneal injections of APAP (100 mg/kg/day) from day 0 starting 30 min prior to receiving the LPS injection, until the end of the study period. Meanwhile, an equivalent volume of vehicle (saline solution) for APAP was given to the CON and LPS groups. The dosage of the drug was chosen according to the results of our preliminary study and is consistent with previous literature sources where the agent was shown to have neuroprotective effects [23, 24].

### Behavioural tests

#### Open field test (OFT)

To evaluate whether the lesioned performance by LPS were attributable to changes in spontaneous locomotor activity, anxiety and adaptivity to the Morris water maze test, open field tests were performed 2 h before the probe test for reference memory. A number of 10 mice in each group (40 mice in total) were randomly selected and subjected to behavioural test in this study. The open field test was performed according to the previous study [30]. The open field apparatus is a black woody box (50 × 50 × 30 cm); the floor was divided into two concentric square shape zones representing the close and far distance to the central of the field. The test was performed under a dark light of 50 lx. Mice were placed in the centre of the open field and allowed a 5-min acclimation period. Their activities were recorded by an overhead video camera. The total moving distance as well as the moving duration in the open field was analysed using animal behavioural tracking system (Smart, San Diego Instruments, San Diego, CA, USA). The open field apparatus was thoroughly cleaned with 5% ethyl alcohol after the test of each mouse and allowed to dry between tests.

#### Morris water maze (MWM)

The MWM test, which is a hippocampal-dependent test of spatial learning, spatial memory and cognitive flexibility for rodents, was performed as described previously with minor modifications [31]. The water maze was a white circular tank made of polyvinyl chloride (118 cm in diameter and 42 cm in height) and filled with white non-toxic paint and water (23 ± 1 °C) to a depth of 28 cm. The maze was placed in a room with several visual cues for orientation in the maze. The maze was divided into four quadrants: the first, second, third and fourth quadrants. An invisible platform (11 cm in diameter) was placed 1 cm below the water surface in the first quadrant (target quadrant). Spatial learning is assessed across repeated trials for 3 days (day −3 to day −1). Mice received one training session consisting of eight trials per day for continuous 3 days (day −3 (training session 1); day −2 (training session 2); day −1 (training session 3)). Mice were released into the water facing the wall of the tank from one of four separate quadrants and were allowed to escape onto the hidden platform. The submerged platform was always located in the first quadrant. A different starting point was used on the eight trials. The mice were trained to find the hidden platform and climb onto it within 60 s. The animals were allowed to stay on the platform for at least 10 s after each trial. When the mice failed to reach the escape platform within 60 s, they were manually guided to the platform for 10 s by the experimenter. After that, the mouse was removed to its cage and the second animal was tested on trial 1. This rotation was repeated until all animals completed trial 1. Subsequently, the process was repeated for subsequent trials until eight trials completed per day for three consecutive days. After each trial, animals were towel dried and returned to their home cage under a heater for 10 min. The animals’ movements were recorded with a video camera attached to the ceiling.

On day 0, animals underwent LPS microinjection. On postoperative day 1, probe tests for reference memory were conducted on all the treated groups by removing the hidden platform and releasing the mice in the third quadrant (opposite to the first quadrant). Swimming speed, platform-site crossings, time travelled in the target quadrant, the percentage of distance travelled in the target quadrant and distance around the platform were recorded in a single 60-s trial. Reference memory was determined by preference for the platform area.

On days 1, 2 and 3, working memory was tested, during which both the platform and mice were randomly placed in a novel position to assess working- or trial-dependent learning and memory [31]. In this procedure, the animal is given two trials per day. On each day, animals underwent the first training trial to ensure that all mice learned the new platform location. After 15 s, the second trial is the test trial or matching trial in which each mouse was released from the same location as in the first trial; if it recalled the first trial, the mice would swim a shorter path to the platform in the second trial. As the platform is moved daily, learning of platform position from the previous day cannot be transferred to the next day; hence, recall on each day during the second trial is dependent on that day’s training trial and measures only temporary or working memory. Measurement of data on the second trial was conducted up to 60 s and the latency for mice that could not reach the platform in the allotted time was regarded as 60 s. Eventually, the escape latency to the platform in the second trial was recorded as measure of temporary or working memory.

### Tissue sampling

At 6, 24, 48 and 72 h after administration of LPS, the mice were sacrificed by cervical decapitation under deep pentobarbital sodium anesthesia. Transcardial perfusion was performed with ice-cold standard phosphate-buffered saline (PBS). The brains of six mice sacrificed at 24 h in each group were quickly removed and fixed in 4% paraformaldehyde for 48 h for histological analysis. Then, the other brains were immediately removed and washed in ice-cold saline. The hippocampus was dissected and collected carefully in a sterile tube prior to being snap-frozen in liquid nitrogen. The hippocampi were stored at −80 °C until analysis. Hippocampi of mice killed at 6 h were used for an enzyme-linked immunosorbent assay (ELISA) for measuring inflammatory cytokines. As previously recorded [32–35], LPS activates microglia and induces pro-inflammatory protein secretion within 6 h in the mouse hippocampus via the NF-κB pathway. All other tests were carried out on post-administration day 1, which corresponded to the time point of the peak behavioural deficits.

### ELISA

Concentrations of interleukin-1β (IL-1β), interleukin-6 (IL-6), and tumour necrosis factor α (TNF-α) were examined using an ELISA kit following the manufacturer’s instructions (Biosource, Invitrogen, USA). Hippocampal tissue was homogenized in RIPA lysis buffer (50 mmol/L Tris–HCl, pH 6.8, 150 mmol/L NaCl, 5 mmol/L EDTA, 0.5% sodium deoxycholate, 0.5% NP-40) and supplemented with a cocktail containing protease and phosphatase inhibitors (Applygen, Beijing, China) on ice. Supernatant protein concentrations were determined after centrifugation at 12,000 rpm for 30 min with a BCA Protein Assay reagent kit (Thermo Pierce, Rockford, IL, USA). For each sample, 5 μL of extracted protein was used for detection. The procedure followed the manufacturer’s instructions. The absorbance was read on a spectrophotometer at a wavelength of 450 nm and a reference wavelength of 650 nm. The concentrations of IL-1β, IL-6 and TNF-α were calculated according to the standard curve and presented as pg/mg protein.

### Immunohistochemistry staining

A cerebral block containing the hippocampus and prefrontal cortex was fixed in 10% neutral-buffered formalin overnight and then embedded in paraffin. Coronal 10-μm sections were prepared and subjected to immunohistochemistry staining. First, paraffin sections were dewaxed and placed in EDTA buffer (pH 8.0) to repair antigens. Second, sections were washed in 0.01% Triton X-100 in phosphate-buffered saline (PBS-T) and blocked with 3% bovine serum albumin (BSA) for 30 min at room temperature. Then, they were incubated overnight at 4 °C in the appropriate primary antibody, anti-Iba1 (1:100; WAKO). Next, the sections were incubated with the appropriate secondary antibody, anti-rabbit IgG (1:400; Jackson) for 2 h at room temperature. Glial reactivity is characterized by an increase in the number of cells and an alteration in cell morphology (rounding of the cell bodies and thickening of processes), which leads to an increase in Iba1 (ionized calcium-binding adaptor molecule 1) labelling with increasing glial reactivity. An increase in the integrated intensity/pixel area for Iba1 staining was interpreted to signify microglial reactivity. The number of positively stained microglial cells per view was counted using microscopy at ×200 magnification. Images were captured using the Olympus BX5 imaging system and quantified using Image-Pro Plus 6.0 software.

### Biochemical analysis

#### Malondialdehyde (MDA)

MDA is a degraded oxidative lipid product from cell membranes and is used as a reliable indicator of oxidative stress [36]. The amount of MDA was measured by the reaction of one molecule of MDA with two molecules of TBA to yield a pink coloured chromogen. The colour reaction was measured at 532 nm with a reference wavelength at 450 nm. The levels of MDA in the hippocampi of mice were measured using commercial assay kits (Beyotime Biotechnology Institute, Nantong, China) according to the manufacturer’s instructions.

#### Superoxide dismutase (SOD) activity

SOD is an endogenous scavenger of reactive oxygen species (ROS) and is one of the major antioxidant enzyme involved in protecting the nerve tissue from oxidative stress. The activity of SOD was measured by the reaction of NBT (nitro blue tetrazolium) with two molecules of superoxide anion to yield a blue coloured chromogen, and SOD has the ability to inhibit the superoxide anion free radical O_2_^−^. The colour reaction was measured at 560 nm with a reference wavelength at 650 nm. The SOD activity of tissue was also measured using commercial assay kits (Beyotime Biotechnology Institute, Nantong, China) according to the manufacturer’s instructions.

### Western blot analysis

Hippocampal tissue was homogenized in RIPA lysis buffer (50 mmol/L Tris–HCl, pH 6.8, 150 mmol/L NaCl, 5 mmol/L EDTA, 0.5% sodium deoxycholate, 0.5% NP-40) plus protease inhibitor and phosphatase inhibitor cocktail (Applygen, Beijing, China) on ice. Supernatant protein concentrations were determined after centrifugation at 12,000 rpm for 30 min with a BCA Protein Assay reagent kit (Thermo Pierce, Rockford, IL, USA). Equal amount of the sample (40 μg of protein) was separated on gradient sodium dodecyl sulphate–polyacrylamide gels (SDS–PAGE) and transferred onto a polyvinylidenedifluoride (PVDF) membrane, which was then blocked with 5% skimmed milk solution. Afterward, the membranes were incubated with primary antibodies overnight at 4 °C. The primary antibodies used in this study were rabbit anti-p-GSK-3β (Ser9) (1:1000; Cell Signaling Technologies), rabbit anti-GSK-3β (1:1000; Cell Signaling Technologies), rabbit anti-Bcl-2 (1:1000; Sigma-Aldrich), mouse anti-Bax (1:50 Sigma-Aldrich), rabbit anti-brain-derived neurotrophic factor (BDNF) IgG (1:1000; Abcam) and mouse anti-β-actin IgG (1:3000; Santa Cruz Biotechnology). After three washes with TBST buffer, the membranes were incubated with goat anti-mouse HRP or goat anti-rabbit HRP (1:3000; Santa Cruz, Biotechnology) for 30 min each. The images were digitized from the membrane, and the band intensity was quantified using Gel-Pro Analyzer software, version 3.1 (Media Cybernetics, Bethesda, MD, USA).

### Apoptosis assay

TUNEL assay was performed to analyse cell death according to the manufacturer’s instructions using In Situ Apoptosis Detection Kit, POD (Roche Applied Science, Mannheim, Germany). The brain tissues including hippocampal cornuammonis (CA) 1, CA3 and dentate gyrus (DG) were harvested, immersed in 3% H_2_O_2_ and washed with phosphate-buffered saline (PBS), incubated with proteinase K solution (Life Technologies, Ambion) at 37 °C for 20 min. Then, the sections were maintained in TUNEL reaction mixture for 1 h at 37 °C, followed by incubation in 50 μL converter POD for another 30 min at 37 °C. After washing with PBS, the sections were incubated with diaminobenzidine (DAB) substrate solution for 10 min. Finally, images were taken using an Olympus BX5 imaging system (Olympus America, Melville, NY, USA) at ×100 and ×400 magnification. The number of apoptotic neurons per view was counted using microscopy at ×400 magnification.

### Statistical analysis

All data were analysed by an observer who was blind to the experimental protocol. Statistical calculations were performed using the statistical analysis software GraphPad Prism, version 6.0 (GraphPad, San Diego, CA, USA). Data were expressed as the mean ± standard error (SE). Intergroup comparisons were conducted by two-way ANOVA (LPS × APAP) followed by Tukey’s post hoc test to determine significant differences between the experimental group. For acquisition training (days −3 to −1) and spatial working memory testing (days 1 to 3) of the MWM, data were analysed using a two-way ANOVA (treatment × trial time) with repeated measures (trial days) followed by a Bonferroni post hoc test to analyse the difference in escape latency between each group. *P* values <0.05 were considered statistically significant.

## Results

### Spontaneous locomotor activity was not changed 24 h after LPS administration

**Fig. 1:**
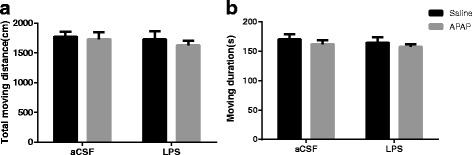
Determine the effect of LPS and APAP on mice in the open field test. The results of spontaneous locomotor activity showing that neither LPS nor APAP changed the total moving distance (**a**) or moving duration (**b**) 24 h after LPS administration. Data are expressed as the mean ± SE (*n* = 10). *APAP* acetaminophen, *aCSF* artificial cerebrospinal fluid, *LPS* lipopolysaccharide, *SE* standard error

To evaluate whether the changes in performance after LPS administration were attributable to changes in spontaneous locomotor activity, the open field test was conducted [37]. No significant difference was observed in locomotor performance (total moving distance and moving duration) 24 h after LPS treatment between the animals in the LPS, A + L, CON and APAP groups (Fig. 1a, b), suggesting that the impaired performance in the LPS group was not a result of reduced locomotor ability.

### APAP attenuated LPS administration-induced learning and memory impairment

**Fig. 2:**
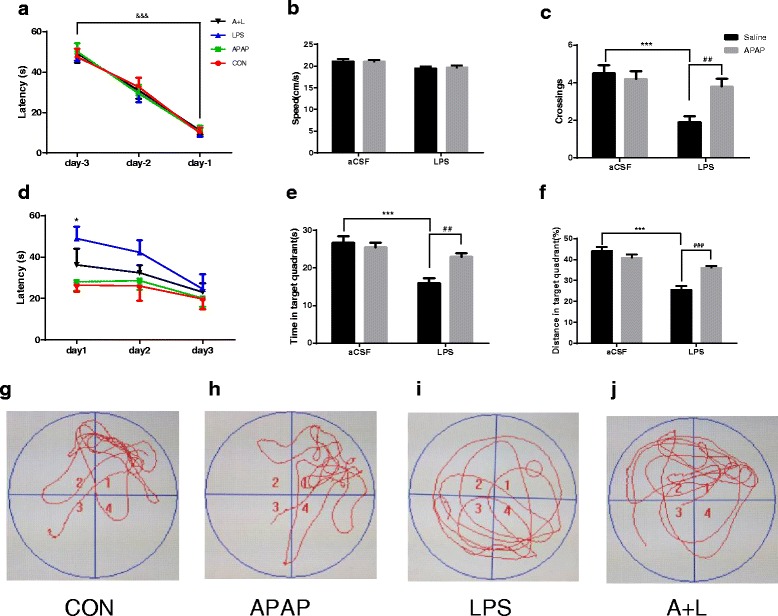
APAP attenuated behavioural performance after exposure to LPS in mice. **a** Spatial learning in the MWM on days −3, −2 and −1. Average escape latency (s) is shown for the three training sessions in the maze. **b** Swimming speed during probe testing on day 1. **c** Platform-site crossings during probe testing on day 1. **e** Time travelled in the target quadrant during probe testing on day 1. **f** Percentage of distance travelled in the target quadrant during probe testing on day 1. **d** Latency to the platform during spatial working memory testing on days 1, 2 and 3. **g**–**j** Representative trajectories of mice from each experimental group during probe testing in which the hidden platform was removed. The *circle* represents the previous location of the platform. Data are expressed as the mean ± SE (*n* = 20). *P < 0.05, ***P < 0.001 vs. CON group; ^##^ *P* < 0.01, ^###^ *P* < 0.001 vs. LPS group; &&&*P* < 0.001 vs. training session 1 in day −3. *APAP* acetaminophen, *aCSF* artificial cerebrospinal fluid, *LPS* lipopolysaccharide, *SE* standard error

Previous work in our laboratory has demonstrated that intracerebroventricular administration of LPS leads to learning and memory deficits [19]. Therefore, the protective effects of APAP on LPS-induced cognitive deficits were examined in this model. As shown in Fig. 2a, in the MWM test, the escape latency in all groups improved over time and was significantly shorter during the third training session than in the first training session (*P* < 0.001), yet no difference was observed between the groups during the same day, indicating that all animals were able to learn where the platform was located. On day 0, animals underwent microinjection surgery, and on postoperative day 1, mice were subjected to a probe test for reference memory, during which the platform was absent. In the probe test, there were no significant differences in swimming speed between groups, suggesting again that the poorer performance of the LPS group was not a result of reduced locomotor ability (Fig. 2b). It was observed that APAP prevented memory deficits caused by LPS on postoperative day 1 (*F*_LPS_ = 14.42, *F*_APAP_ = 4.101, *F*_LPS × APAP_ = 7.754 for platform-site crossings, *P* < 0.05; *F*_LPS_ = 25.43, *F*_APAP_ = 4.799, *F*_LPS × APAP_ = 9.700 for time travelled in the target quadrant, *P* < 0.05; *F*_LPS_ = 50.43, *F*_APAP_ = 4.876, *F*_LPS × APAP_ = 17.53 for percentage of distance travelled in the target quadrant during probe testing, *P* < 0.05; Fig. 2c, e, f). On days 1 to 3, mice were subjected to a working memory test, during which both the platform and mice were randomly placed in a novel position to assess working- or trial-dependent learning and memory. If the animal recalls the sample trial, it will swim a shorter path to the goal on the second trial. On day 1, it was observed that the escape latency needed to reached the new platform was significantly higher (*P* < 0.05) in the LPS group than in the CON and APAP group, and there was no significant difference in escape latency between the A + L group and the CON and APAP group, although mice in the A + L group exhibited a tendency towards an increase in escape latency (Fig. 2d). On day 2, the escape latency of the LPS group still showed tendency for a higher latency than that of the other groups, although it did not reach statistical significance (Fig. 2d). On day 3, the impaired performance of the LPS group in the working memory test returned to control levels, and there was no significant difference in escape latency among all groups (Fig. 2d).

Collectively, our results suggest that LPS causes cognitive impairment, specifically a deficit in short-term memory retention, which can be ameliorated by APAP treatment.

### APAP suppressed the accumulation of pro-inflammatory cytokines induced by LPS in the mouse hippocampus

**Fig. 3:**
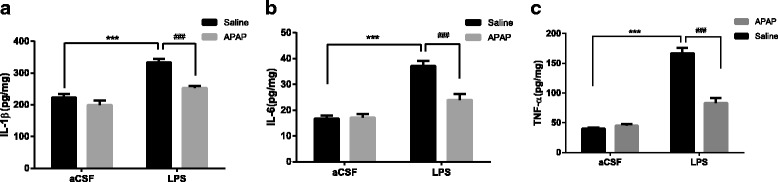
APAP attenuated the accumulation of pro-inflammatory cytokines induced by LPS in the mouse hippocampus. Levels of IL-1β (**a**), IL-6 (**b**) and TNF-α (**c**) in samples of the hippocampus 6 h after LPS administration. IL-1β, IL-6 and TNF-α were increased by LPS, and APAP reversed the accumulation of those pro-inflammation cytokines. Data are expressed as the mean ± SE (*n* = 5, 6) ***P < 0.001 vs. CON group; ^###^ *P* < 0.001 vs. LPS group. *APAP* acetaminophen, *aCSF* artificial cerebrospinal fluid, *LPS* lipopolysaccharide, *SE* standard error

We investigated the levels of several pro-inflammatory cytokines (IL-1β, IL-6 and TNF-α) in the hippocampus, a brain region where neuroinflammation mainly occurs in response to brain injury and inflammation [38, 39]. In accordance with our previous study [19], an ELISA immunoassay showed that the level of IL-1β (*F*_LPS_ = 60.95, *P* < 0.001, Fig. 3a), IL-6 (*F*_LPS_ = 53.98, *P* < 0.001, Fig. 3b) and TNF-α (*F*_LPS_ = 163.6, *P* < 0.001, Fig. 3c) in the LPS group was much higher than that in the CON group. Remarkably, APAP significantly attenuated LPS-induced increases in IL-1β (*F*_APAP_ = 25.65, *P* < 0.001, Fig. 3a), IL-6 (*F*_APAP_ = 11.61, *P* < 0.001, Fig. 3b) and TNF-α (*F*_APAP_ = 37.92, *P* < 0.001, Fig. 3c) levels. These results suggest that APAP inhibited LPS-induced accumulation of pro-inflammatory cytokines in the mouse hippocampus.

### APAP prevented microglial activation after intracerebroventricular administration of LPS

**Fig. 4:**
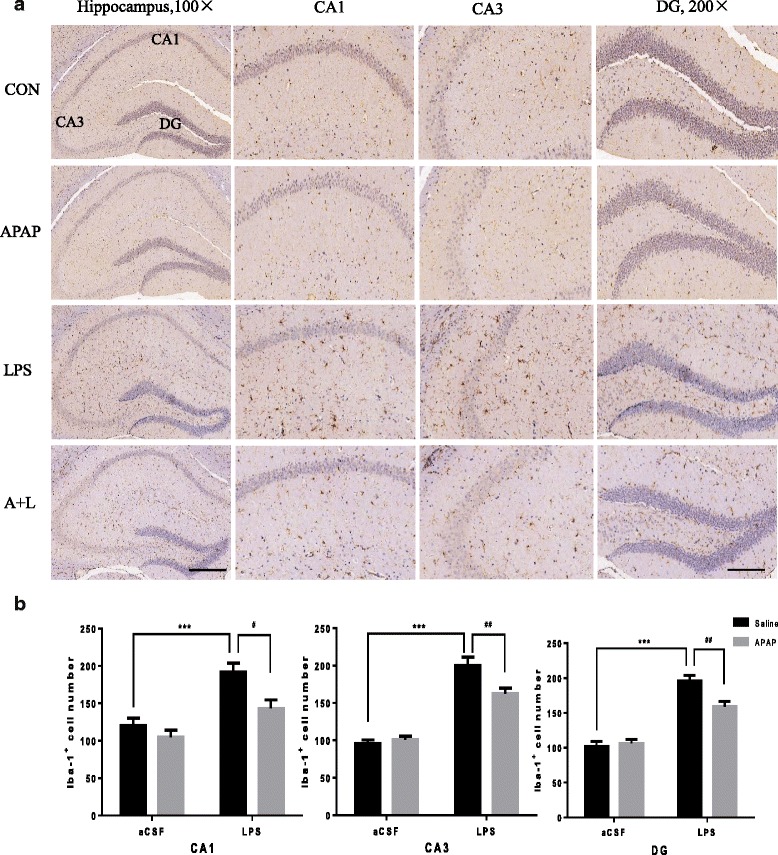
APAP attenuated LPS-induced microglial (*brown*) activation. **a** Representative images of Iba1-labelled activated microglia in the hippocampi 24 h after LPS administration. **a** Activated microglia and cell nuclei in the hippocampi on postoperative day 1. **b** The number of hippocampal Iba1-positive cells on postoperative day 1 under ×200 magnification. *Scale bars*: ×100, 200 μm; ×200, 100 μm. Data are expressed as the mean ± SE (*n* = 5–6). ***P < 0.001 vs. CON group; ^#^ *P* < 0.05, ^##^ *P* < 0.01 vs. LPS group. *APAP* acetaminophen, *aCSF* artificial cerebrospinal fluid, *LPS* lipopolysaccharide, *SE* standard error

Due to the important role that cytokines and microglial activation play in LPS-induced neuroinflammation [14, 40–42], we used immunohistochemistry to investigate microglial activation. The immunohistochemistry results showed that LPS caused obvious microglial activation in the mouse hippocampal DG regions (*F*_LPS_ = 109.7, *P* < 0.001), CA1 regions (*F*_LPS_ = 26.93, *P* < 0.001) and CA3 regions (*F*_LPS_ = 135.1, *P* < 0.001) labelled by Iba1 (Fig. 4b). APAP significantly attenuated LPS-induced microglial activation (*F*_APAP_ = 5.547 and *P* = 0.0211, *F*_APAP_ = 9.321 and *P* = 0.0042 and *F*_APAP_ = 5.098 and *P* = 0.0301 for DG regions, CA1 regions and CA3 regions, respectively) in the hippocampus of mice (Fig. 4b).

### APAP increased SOD activity and reduced MDA levels in the hippocampi of mice after LPS administration

**Fig. 5:**
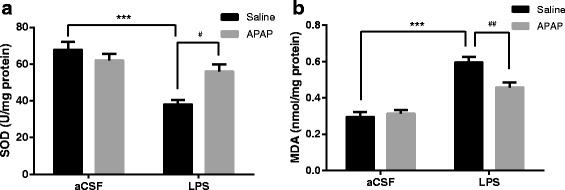
APAP increased SOD activity and reduced hippocampal MDA levels after exposure to LPS in mice. **a** SOD activity in the hippocampus on postoperative day 1. **b** The MDA level in the hippocampus on postoperative day 1. Data are expressed as the mean ± SE (*n* = 6). ***P < 0.001 vs. CON group; ^#^ *P* < 0.05, ^##^ *P* < 0.01 vs. LPS group. *SOD* superoxide dismutase, *MDA* malondialdehyde, *APAP* acetaminophen, *aCSF* artificial cerebrospinal fluid, *LPS* lipopolysaccharide, *SE* standard error

As shown in Fig. 5a, compared to the CON group, LPS induced a significant decrease in SOD activity in the hippocampus on postoperative day 1 (*F*_LPS_ = 24.53, *P* < 0.001, *n* = 6); this abnormal decrease in SOD activity was largely prevented by APAP (*F*_APAP_ = 2.737, *P* = 0.0111, *n* = 6). As demonstrated in Fig. 5b, the MDA level of the hippocampi in the LPS group was significantly higher on postoperative day 1 than that in the CON group (*F*_LPS_ = 67.35, *P* < 0.001, *n* = 6). Likewise, APAP significantly attenuated abnormally increased MDA levels in the hippocampi of the A + L group (*F*_APAP_ = 4.796, *P* = 0.0099, *n* = 6). Meanwhile, no significant difference was found between the APAP alone and CON groups.

### APAP blocked glycogen synthase kinase 3β (GSK3β) activity and attenuated the BDNF decrease in mice with LPS-induced hippocampal impairment

**Fig. 6:**
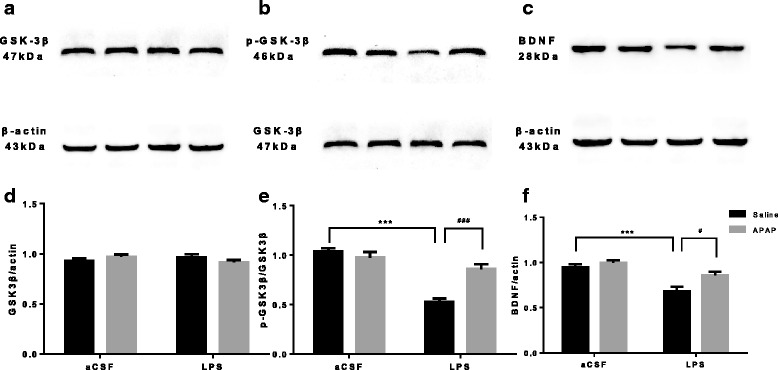
Effect of APAP on the expression of p-GSK3β/GSK3β and BDNF in mice with hippocampal impairment. **a**–**c** Protein bands on the gel and their relative intensities on postoperative day 1. **d**–**f** The expression levels of p-GSK3β/GSK3β and BDNF proteins were normalized to that of β-actin as an internal control. Data are expressed as the mean ± SE (*n* = 6). ***P < 0.001 vs. CON group; ^#^ *P* < 0.05, ^###^ *P* < 0.001 vs. LPS group. *APAP* acetaminophen, *aCSF* artificial cerebrospinal fluid, *LPS* lipopolysaccharide, *SE* standard error

GSK3β is an important kinase that was first identified as the major regulator of glycogen synthase, the key enzyme involved in glycogen synthesis. However, similar to many other kinases, GSK3β was found to regulate numerous other processes, including those involving cell death, apoptosis and cognitive function [43, 44]. Therefore, the present experiments were designed to determine whether APAP is able to modulate GSK3β activity in the LPS-exposed mouse hippocampus. The total expression level of GSK3β was not altered by different treatments (*F*_LPS_ = 0.17, *P* > 0.05; *F*_APAP_ = 0.01257, *P* > 0.05; Fig. 6d), whereas the phospho-GSK3β/GSK3β ratio in the hippocampus of LPS-exposed mice was significantly decreased, and APAP attenuated this reduction (*F*_LPS_ = 48.37, *P* < 0.001; *F*_APAP_ = 8.867, *P* = 0.0074; Fig. 6e). These data suggest that APAP treatment downregulates GSK3β activity by phosphorylating GSK3β in the hippocampus. In the LPS-treated hippocampus, total GSK3β remained unchanged, and the activation of GSK3β was relatively higher than in the CON group.

BDNF can enhance protective pathways and inhibit damaging pathways, supporting the survival of existing neurons and promoting neurogenesis [45, 46]. The effects of LPS and APAP on BDNF were evaluated. The expression of BDNF was reduced in the LPS-treated group, which was alleviated by APAP (*F*_LPS_ = 25.73, *P* < 0.001; *F*_APAP_ = 7.706, *P* = 0.0117; Fig. 6f), suggesting that APAP may protect hippocampal neurons from LPS-induced damage.

### APAP significantly decreased the Bax/Bcl-2 ratio and neuron apoptosis in the hippocampus of LPS-treated mice

**Fig. 7:**
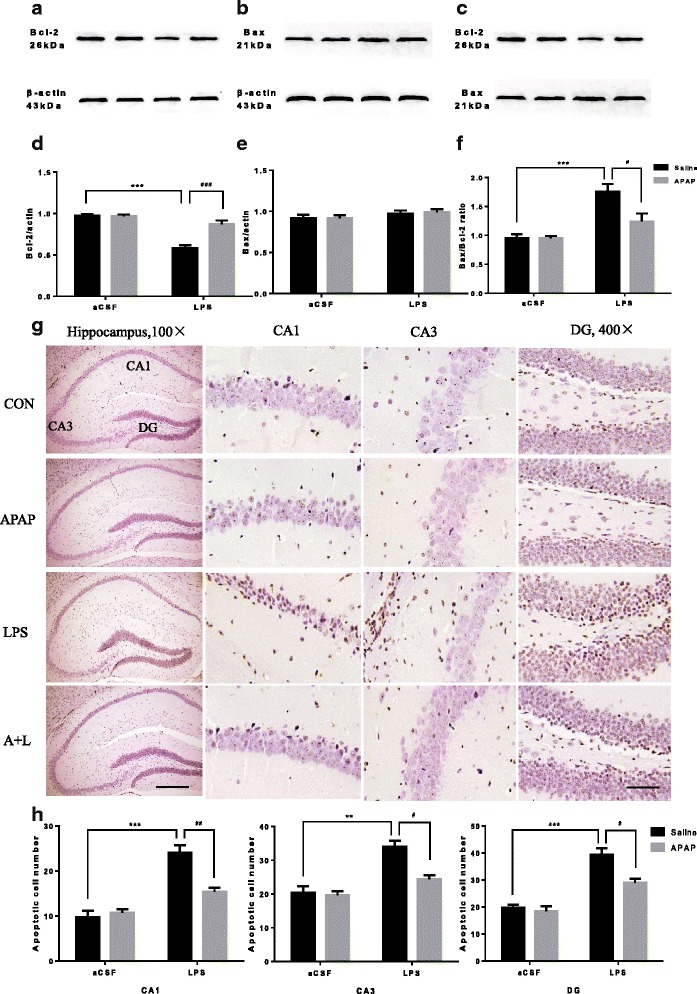
Effect of APAP on the expression of Bax/Bcl-2 and neuron apoptosis in mice with hippocampal impairment. **a**–**c** Protein bands on the gel and their relative intensities on postoperative day 1. **d**–**f** The expression levels of Bax/Bcl-2 proteins were normalized to that of β-actin as an internal control. **g** CA1, CA3 and DG regions of hippocampal from indicated group of mice. TUNEL staining demonstrated neuron apoptosis on postoperative day 1. **h** The number of hippocampal apoptotic neurons on postoperative day 1 under ×400 magnification. *Scale bars*: ×100, 200 μm; ×400, 50 μm. Data are expressed as the mean ± SE (*n* = 6). **P < 0.01, ***P < 0.001 vs. CON group; ^#^ *P* < 0.05, ^##^ *P* < 0.01, ^###^ *P* < 0.001 vs. LPS group. *APAP* acetaminophen, *aCSF* artificial cerebrospinal fluid, *LPS* lipopolysaccharide, *SE* standard error

To assess whether the balance of hippocampal pro-apoptotic protein Bax and anti-apoptotic protein Bcl-2 was affected by LPS, the expression of Bax and Bcl-2 proteins was measured using immunoblotting. As shown in Fig. 7d, the expression of Bcl-2 protein was significantly decreased in the LPS-treated mice (*F*_LPS_ = 55.54, *P* < 0.001, *n* = 6), whereas APAP treatment was able to restore Bcl-2 protein content to that comparable to the CON group (*F*_APAP_ = 19.51, *P* < 0.001, *n* = 6). The expression of Bax protein was not different among the four groups (*F*_LPS_ = 2.37, *F*_APAP_ = 0.062, *P* > 0.05, Fig. 7e). Further analysis of the Bax/Bcl-2 ratio, an index of pro-apoptotic signalling pathway activation [47], demonstrated that the Bax/Bcl-2 ratio was higher in the LPS group than in the CON group (*F*_LPS_ = 27.98, *P* < 0.001, *n* = 6, Fig. 7f). APAP treatment significantly decreased the Bax/Bcl-2 ratio in the hippocampi of the A + L group compared to the LPS group (*F*_APAP_ = 6.140, *P* = 0.0223, *n* = 6, Fig. 7f). In order to observe the effect of APAP on LPS-induced hippocampal neurons, we carried out TUNEL assay. We found that the apoptotic cell number of the DG, CA1 and CA3 regions in the LPS group was significantly higher than that in the CON group (*P* < 0.001 for DG regions, *P* < 0.001 for CA1 regions and *P* = 0.0013 for CA3 regions, respectively, Fig. 7h). However, APAP significantly attenuated LPS-induced neuron apoptosis in the hippocampal DG, CA1 and CA3 regions (*P* = 0.0145 for DG regions, *P* = 0.0063 for CA1 regions and *P* = 0.0109 for CA3 regions, respectively, Fig. 7h). Our results suggested that APAP may ameliorate the learning and memory ability by reducing LPS-induced hippocampal neuron apoptosis.

## Discussion

This paper shows that APAP improved cognitive dysfunction induced by cerebroventricular administration of LPS. The protective effects of APAP on cognitive impairment in mice may be related to its antioxidant and anti-inflammatory activity, modulating the activity of GSK3β, as well as its ability to inhibit the mitochondrial permeability transition (MPT) pore and the subsequent apoptotic pathway.

Recent experimental data suggest that APAP may have several remarkable effects other than its well-known analgesic/antipyretic properties. Thus far, a wide array of neuroprotective effects of APAP has been reported in different studies [24, 27–29, 48]. Previous studies have shown that APAP can protect neurons from degeneration in animal models and cell lines of AD [29] and PD [49] that involves inflammatory and oxidative stress processes. Given the evidence that sepsis can cause cognitive impairment in human subjects [50, 51], our study focused on the effects of APAP in an LPS-induced cognitive impairment mouse model.

The MWM test was chosen as a robust and reliable test that is strongly correlated with hippocampal-dependent memory [52, 53]. Escape from water is relatively immune from activity or body mass differences, making it ideal for experimental models. As previously reported [19], there were no significant differences between groups during the acquisition phase, and 2 μg of LPS (intracerebroventricular administration) caused memory deficits on postoperative day 1 following the microinjection. In the preliminary experiment, mice treated with 2 μg of LPS did not remember where the platform was and searched aimlessly instead. We found that 100 mg/kg of APAP ameliorated the memory deficits caused by LPS. Similar results were obtained in formal experiments using the spatial reference memory test; an obvious inherent memory impairment was observed in the LPS group, and this inherent memory injury was significantly alleviated by APAP. In the MWM trail-dependent learning task, an obvious impairment in working memory was observed in the LPS group, and this working memory injury was alleviated by APAP although there was no statistical significance. Collectively, our results indicate that LPS causes cognitive impairment, specifically a deficit in short-term memory retention, which can be ameliorated by APAP treatment. This finding suggests a potential application of APAP in patients with or at risk for cognitive impairment. The results of the current study are consistent with a previous study that showed that administration of APAP improves cognitive performance of rodents in the Morris water maze test [48].

Considerable evidence implicates neuroinflammation in the pathophysiology of progressive neurodegenerative disorders such as AD, PD, ALS and MS [5–7]. A link between increased cytokine formation and neurodegeneration has been demonstrated [54]. Thus, we next evaluated the effect of APAP on neuroinflammatory processes induced by LPS administration. Microglia is the resident macrophage in the brain, participates in the coordination of events important for the maintenance of neuronal health and plays the most important role in responding to inflammation in the central nervous system. Previous studies [32–35, 41] have shown that LPS triggers microglia activation and consequently induces pro-inflammatory cytokine secretion within 6 h via the NF-κB pathway in the mouse hippocampus and that the nuclear signal of the transcription factor is strongly reduced or blocked by anti-inflammatory compounds [55, 56]. In the current study, we demonstrated that intracerebroventricular injection of LPS induced increases in TNF-α, IL-1β and IL-6 levels in the mouse brain 6 h after the injection. In addition, microglia labelled by Iba1 in the hippocampus were activated. The current studies showed that APAP inhibited LPS-induced microglial activation and production of pro-inflammatory mediators including TNF-α, IL-1β and IL-6. Our results are in accordance with previous reports that APAP blunted neuronal apoptosis via reduction of the inflammatory transcription factor NF-κB and reduces inflammatory protein such as chemokines and cytokines [57]. APAP has the capability to modulate the release of inflammatory molecules such as PGE2 and IL-6 by Aβ-stimulated astrocytes [58]. APAP also protects brain endothelial and neuronal cells against oxidative stress, and menadione-induced increase in chemokines and cytokines was reduced by APAP [25, 26]. Thus, APAP possesses the ability to block NF-κB activation and exert a neuroprotective effect, suggesting that the neuroprotective effects of APAP might be related to its anti-neuroinflammation effects.

Oxidative stress is defined as an imbalance between higher cellular levels of reactive oxygen species (ROS), e.g. superoxide and hydroxyl radicals [59], and cellular antioxidant defence [60]. If ROS are not controlled by enzymatic and non-enzymatic antioxidants, they can cause oxidative injury, i.e. peroxidation of cell membrane phospholipids, proteins (receptors and enzymes) and DNA. The brain is extremely susceptible to oxidative damage induced by ROS because it generates very high levels of ROS due to its very high aerobic metabolism and blood perfusion and its relatively poor enzymatic antioxidant defence [61]. The brain contains polyunsaturated fatty acids (PUFAs), which can readily be peroxidized [62]. Lipid peroxidation (LP) causes injury to cells and intracellular membranes and may lead to cell destruction and subsequently cell death [59, 63]. Therefore, oxidative stress participates in neuronal injury and cognitive impairment. However, increasing evidence suggests that APAP has unappreciated antioxidant properties. For example, APAP can protect dopaminergic neurons from 1-methyl-4-phenylpyridinium (MPP^+^)-induced toxicity in mitochondria by scavenging ROS [23]. Additionally, administration of APAP to rats significantly attenuates quinolinic acid-induced superoxide generation [24]. APAP has been shown to be a potent scavenger of ROS [26]. Previous studies have indicated that after intracerebroventricular injection of LPS, significant changes in oxidative stress markers were found in the hippocampus of mice [19]. Indeed, decreased activity of SOD and increased levels of MDA were found after LPS microinjection in the hippocampi of animals in the LPS group. Abnormal changes in the activity of SOD and the levels of MDA were partially reversed by APAP, suggesting that the neuroprotective effects of APAP might be related to its antioxidant effects.

GSK3β regulates many crucial cellular processes in the brain and plays an important role in the inflammatory process [64, 65]. GSK-3β overexpression induces apoptosis and causes a reduction in postsynaptic density number and volume in hippocampal granule neurons [66], a phenomenon that may be related to cognitive impairment. GSK3β is constitutively active in the cytoplasm, but its activity can be inhibited by phosphorylation of protein kinase B (Akt) at the site of serine 9 [67]. Our results have shown that intracerebroventricular LPS depressed the phospho-GSK3β/GSK3β ratio in the hippocampus, and dysregulation of this signal transduction pathway could result in failure to adequately repress GSK-3β, thus allowing GSK-3β to remain abnormally active. This status has been proven to contribute to various pathologies, including neurodegenerative and cognitive disorders, which is in line with behavioural performance [65, 68]. However, APAP attenuated the reduction by phosphorylating GSK3β in the hippocampus and blocked GSK-3β activity in response to LPS, thus providing protection from LPS-induced apoptosis and cognitive impairment. This observation might be caused by the antioxidant effects of APAP. It is known that oxidative stress can activate GSK3β by Src- and calcium-dependent mechanisms [69]. However, recent studies have shown that APAP could modulate microsomal pores, rectify Ca^2+^ homeostasis and, therefore, decrease ROS production in the rat hippocampus [70].

BDNF and its signalling pathways are firmly implicated in neuronal differentiation and survival. Numerous pieces of evidence indicate that BDNF regulates both early and late phases of long-term potentiation in the hippocampus [71]. Its regulation of synaptic plasticity may underlie hippocampus-dependent learning and memory. Most studies [17, 72–74], including ours, agreed that LPS injections extensively reduced the expression of BDNF in the brain. This decline was altered by APAP treatment in our study. The underlying mechanism for this effect might be that the active GSK3β, caused by LPS injection, phosphorylates the cyclic adenosine monophosphate (cAMP) response element-binding protein (CREB) and could thereby regulate transcription of genes related to synaptic plasticity and neurogenesis, including BDNF [75]. As our results showed, APAP blocked GSK-3β activity in mice with LPS-induced hippocampal impairment. Therefore, the decrease in BDNF was subsequently reversed.

It is well known that LPS causes cognitive lesions and that this process involves apoptosis [76, 77]. A large number of proteins operate in concert to regulate apoptosis. Bcl-2 is of particular interest as it has been reported to be protective against oxidative stress, reducing cell death induced by reactive oxygen species [78–80]. Previous studies have shown that cells overexpressing anti-apoptotic Bcl-2 exhibit a marked reduction in apoptosis, which suggests that anti-apoptotic Bcl-2 could independently affect the rate of apoptosis [81]. Anti-apoptotic Bcl-2 family members can attenuate the release of killer proteins, influence mitochondrial integrity and prevent apoptosis [82, 83]. Similar studies employing neuronal cells modelling protein aggregation typical of Alzheimer’s disease have also reported a significant reduction in apoptotic cell death when these cells overexpressed anti-apoptotic Bcl-2 [84]. Recent in vitro data have shown that APAP increases expression of anti-apoptotic protein Bcl-2 in brain endothelial cells and neuronal cells experiencing oxidative stress as a by-product of inflammation [25, 26]. In this paper, our results are consistent with previous studies; we found that LPS-induced cognitive impairment was associated with decreased Bcl-2 expression and an elevated Bax/Bcl-2 ratio and that APAP protects against apoptosis, thereby assuaging cognitive impairment, by increasing the expression of anti-apoptotic Bcl-2 proteins and decreasing the hippocampal neuron apoptosis. APAP is likely to prevent mitochondrial pore dysfunction, resulting in the stabilization of pro-apoptotic proteins and consequently minimizing their deleterious effects on MPT, which precede apoptosis and necrosis [83].

## Conclusions

In summary, obvious cognitive impairment was shown in mice that underwent intracerebroventricular microinjection of LPS. Our in vivo studies showed that APAP could alleviate cognitive impairment induced by LPS. Its protective mechanism may be related to its antioxidant and anti-inflammatory properties and modulation of GSK3β activity, as well as its ability to inhibit the MPT pore and subsequent apoptotic pathway. Although the molecular mechanisms of action of APAP are controversial due to its hepatotoxicity at high doses [85], further investigation of the neuroprotective activity of APAP in the mammalian system is warranted given the prospect for this common medication to be used for prophylactic, as well as adjuvant, therapy for neurodegenerative diseases resulting from oxidative and inflammatory damage.

## Abbreviations

aCSF: Artificial cerebrospinal fluid
AD: Alzheimer’s disease
ALS: Amyotrophic lateral sclerosis
APAP: Acetaminophen
BDNF: Brain-derived neurotrophic factor
CA: Cornuammonis
DG: Dentate gyrus
ELISA: Enzyme-linked immunosorbent assay
GSK3β: Glycogen synthase kinase 3β
LPS: Lipopolysaccharide
MDA: Malondialdehyde
MPT: Mitochondrial permeability transition
MS: Multiple sclerosis
MWM: Morris water maze
PD: Parkinson’s disease
POCD: Postoperative cognitive dysfunction
SE: Standard error
SOD: Superoxide dismutase
